# Puerperal Fever: Its Definition and Its Notification

**Published:** 1898-12-10

**Authors:** 


### Puerperal Fever: Its Definition and its Notification.

One of the great difficulties in the way of carrying out the provisions of the Notification Act arises from the lack of any definition of certain of the diseases included in its schedule, and we need hardly be surprised that in regard to so ill-defined a disease as puerperal fever notification has been more or less perfunctory, and has led to but little practical advantage.

Who is prepared to tell us what is meant by puerperal fever? If we consult the older text-books we, no doubt, find a fairly distinct clinical picture of a disease which fortunately we but seldom see. The notification of this disease, however—the desperate and rapidly fatal form of puerperal septicæmia—does not content the sanitary authorities, while any definition which will cover all the septic cases occurring after childbirth which they want to have reported will include many cases which undoubtedly are not puerperal fever in the ordinary acceptation of the term. Under these circumstances there was a certain reasonableness in the suggestion made by the vestry of Bethnal Green to the London County Council, that certain well-recognised infective conditions occurring after childbirth should be added to the list of notifiable diseases; such conditions being peritonitis and metritis, occurring in connection with parturition, as well as puerperal pyæmia, puerperal septicæmia, and puerperal sapræmia. The present difficulty circles round the use of the term "puerperal fever," which, according to the Royal College of Physicians, is obsolete, and had already been expunged from their Nomenclature of Diseases before the Notification Act was passed, and it is brought to a head by a magisterial decision to the effect that even puerperal peritonitis is not included in the disease called puerperal fever in Section LV. of the Act. Under these circumstances the London County Council consulted the Royal College of Physicians, and a committee of the College have stated that in their opinion "the London County Council would be acting rightly in adopting the view that the expression 'puerperal fever,' as contained in Section LV. of the Public Health Act, 1891, should be taken to include septicæmia, pyæmia, septic peritonitis, septic metritis, and other acute specific inflammations in the pelvis occurring as the direct result of childbirth." It cannot, however, be held that this definition is in any way binding. It is not pretended, indeed, that it represents what was in the minds of those by whom the Notification Act was framed, and it is quite certain that the vast majority of medical men would entirely deny that a case of, say, acute pelvic abscess, however clearly it might be the "direct result" of childbirth, is a case of "puerperal fever." It is very unfortunate, but there can be but little doubt that the matter will have to be fought out in the Law Courts. Some vestry or another is sure to want to put the law in force, and some medical man or another is almost sure to be found who will differ from the very wide and all-embracing definition given above. Nor will arguments be lacking to those who choose to resist the ruling of the College. The words of the Act are "puerperal fever," and plenty of evidence will be available to show that a septic abscess—such as would be included in the College definition—is not puerperal fever, and, in fact, that one of the first things one has to ascertain, on finding a rise of temperature after a confinement, is whether it is due to such a local septic disturbance, or to the onset of "puerperal fever"—whatever that may mean. There will also be plenty of hairs to split over the word "septic," which the Royal College seems to find so blessed, and probably the Law Courts will have to decide, among other abstruse questions, whether the bacillus coli is, or is not, a "septic" organism. If the matter were not one which is likely to lead to many complications we should not give to it so much importance. But we cannot shut our eyes to the fact that the notification of puerperal fever stands on a very different footing from that of all other diseases. The tendency of all writers on midwifery has lately been to lay down with great positiveness the dictum that septic puerperal disease is introduced from without, and, to put it roughly, is the fault of the accoucheur. To ask for notification of this disease is then to a growing extent to ask for information from the medical attendant which may be used against him, if not by the authorities at any rate by the public. This in itself must always throw difficulties in the way of notification, but when we find the definition of the disease so widened as to include such widely separated conditions, and find that medical men who come across cases of local inflammatory conditions are asked to brand their practices with the dreaded words "puerperal fever," we may be pretty sure that they will resist, and that the matter will have to be settled in the Law Courts. On the whole the simplest plan would have been either to have adopted the suggestion of the Bethnal Green Vestry, or to have added to the list of notifiable diseases all acute septic inflammations, other than puerperal fever, occurring as the direct result of childbirth.

